# LIM domain proteins Pinch1/2 regulate chondrogenesis and bone mass in mice

**DOI:** 10.1038/s41413-020-00108-y

**Published:** 2020-10-13

**Authors:** Yiming Lei, Xuekun Fu, Pengyu Li, Sixiong Lin, Qinnan Yan, Yumei Lai, Xin Liu, Yishu Wang, Xiaochun Bai, Chuanju Liu, Di Chen, Xuenong Zou, Xu Cao, Huiling Cao, Guozhi Xiao

**Affiliations:** 1 Guangdong Provincial Key Laboratory of Cell Microenvironment and Disease Research, Shenzhen Key Laboratory of Cell Microenvironment, and School of Medicine, Southern University of Science and Technology, Shenzhen, 518055 China; 2 Department of Spine Surgery, Orthopedic Research Institute, The First Affiliated Hospital of Sun Yat-sen University, Guangdong Provincial Key Laboratory of Orthopedics and Traumatology, Guangzhou, 510080 China; 3 Department of Orthopedic Surgery, Rush University Medical Center, Chicago, IL 60612 USA; 4 Department of Cell Biology, School of Basic Medical Sciences, Southern Medical University, Guangzhou, 510515 China; 5 Department of Orthopedic Surgery, New York University School of Medicine, New York, NY 10003 USA; 6 Department of Cell Biology, New York University School of Medicine, New York, NY 10016 USA; 7 Research Center for Human Tissues and Organs Degeneration, Shenzhen Institutes of Advanced Technology, Chinese Academy of Sciences, Shenzhen, 518055 China; 8 Department of Orthopedic Surgery, The Johns Hopkins University, Baltimore, MD 21205 USA

**Keywords:** Bone, Bone quality and biomechanics

## Abstract

The LIM domain-containing proteins Pinch1/2 regulate integrin activation and cell–extracellular matrix interaction and adhesion. Here, we report that deleting Pinch1 in limb mesenchymal stem cells (MSCs) and Pinch2 globally (double knockout; dKO) in mice causes severe chondrodysplasia, while single mutant mice do not display marked defects. Pinch deletion decreases chondrocyte proliferation, accelerates cell differentiation and disrupts column formation. Pinch loss drastically reduces Smad2/3 protein expression in proliferative zone (PZ) chondrocytes and increases Runx2 and Col10a1 expression in both PZ and hypertrophic zone (HZ) chondrocytes. Pinch loss increases sclerostin and Rankl expression in HZ chondrocytes, reduces bone formation, and increases bone resorption, leading to low bone mass. In vitro studies revealed that Pinch1 and Smad2/3 colocalize in the nuclei of chondrocytes. Through its C-terminal region, Pinch1 interacts with Smad2/3 proteins. Pinch loss increases Smad2/3 ubiquitination and degradation in primary bone marrow stromal cells (BMSCs). Pinch loss reduces TGF-β-induced Smad2/3 phosphorylation and nuclear localization in primary BMSCs. Interestingly, compared to those from single mutant mice, BMSCs from dKO mice express dramatically lower protein levels of β-catenin and Yap1/Taz and display reduced osteogenic but increased adipogenic differentiation capacity. Finally, ablating Pinch1 in chondrocytes and Pinch2 globally causes severe osteopenia with subtle limb shortening. Collectively, our findings demonstrate critical roles for Pinch1/2 and a functional redundancy of both factors in the control of chondrogenesis and bone mass through distinct mechanisms.

## Introduction

In vertebrates, the skeleton is formed through intramembranous and endochondral ossification.^1,2^ The former forms the skull vault and part of the clavicle directly through condensation and differentiation of mesenchymal stem cells (MSCs) into osteoprogenitors, osteoblasts, and, terminally, osteocytes, while the latter forms the majority of skeletal elements, including all long bones and vertebrae. During endochondral ossification, a cartilage anlage is initially formed through a process that involves MSC condensation, chondrocyte proliferation, differentiation, hypertrophy, and apoptosis. The anlage is eventually digested and replaced by bone in the adjacent metaphysis; this process involves new blood vessel invasion, osteoclast differentiation, digestion of the calcified cartilage, osteoblastogenesis from perichondrial cells, and bone formation.^1–3^ Endochondral ossification is critical for the longitudinal growth of the skeleton. Abnormal endochondral ossification causes chondrodysplasia and dwarfism.

A number of factors are required for proper control of chondrogenesis.^2,4^ Among these factors, Sox9, a transcription factor of the sex-determining region Y (SRY)-related high mobility group box family of proteins,^5,6^ acts as a major regulator of chondrogenesis by promoting MSC condensation and chondrocyte formation and proliferation and inhibiting chondrocyte differentiation and hypertrophy.^7–14^ In addition to acting as a master regulator of osteoblast and bone formation,^15–18^ Runx2 directly activates the transcription of the collagen type X alpha 1 chain (*Col10a1*) gene and promotes chondrocyte differentiation and hypertrophy.^19–23^ Transforming growth factor-β (TGF-β) is critical for chondrocyte function and skeletogenesis^24–30^ and represses chondrocyte differentiation and hypertrophy.^31,32^ TGF-β exerts its function primarily through binding to its receptors (TβRI and TβRII), which causes the transactivation of TβRI by TβRII and activation and nuclear translocation of R-Smad (Smad2/3). Interestingly, TGF-β suppresses Runx2 expression and activity.^33^ While the importance of the above factors in the control of skeletogenesis is well documented in the literature, the key signals that modulate their expression and function are incompletely defined.

Mammalian cells have two functional Pinch proteins, Pinch1 (encoded by *Lims1*) and Pinch2 (encoded by *Lims2*). Pinch1/2 are five LIM domain-containing proteins that play important roles in integrin activation, cytoskeletal organization, cell–extracellular matrix adhesion, migration, proliferation, differentiation, and survival.^34–39^ Both Pinch1 and Pinch2 are ubiquitously expressed in most mammalian tissues and organs.^38^ Global deletion of Pinch1 in mice is lethal,^35^ while Pinch2 knockout mice display no apparent phenotypes.^37,40^ Pinch proteins exert their functions in part by forming distinct functional protein–protein complexes, including the ILK–Pinch–Parvin (IPP), Pinch-Nck2, and Pinch-Rsu1 complexes.^36,38,41–44^ The IPP complex regulates cell contractility and cytoskeletal dynamics in mice.^39,45–47^ Previous studies on Pinch proteins have primarily focused on their roles in cancers, such as tumor cell growth, apoptosis, progression, invasion, and radio- and chemoresistance.^37,38,48–51^ However, the roles of Pinch proteins in skeletogenesis have not been established.

The aim of this study was to investigate whether Pinch1/2 play roles in skeletogenesis and, if so, to determine the underlying mechanisms. We evaluated the effects of deleting Pinch1 in limb MSCs using *Prx1-Cre* mice or in chondrocytes using *Col2a1-Cre* mice and/or deleting Pinch2 globally in mice. Through comprehensive analyses of cells and tissues from multiple genetic mouse models, we established critical roles for Pinch1/2 and a functional redundancy of both factors in the control of chondrogenesis and bone mass through distinct mechanisms.

## Results

### Double knockout (dKO), but not single mutant, mice display dwarfism and severe osteopenia

**Fig. 1:**
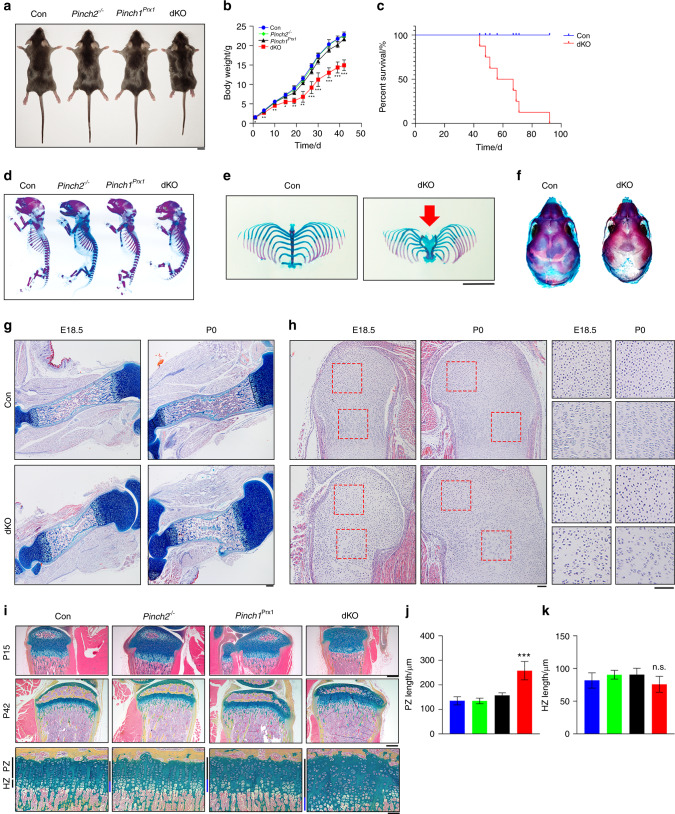
*Pinch1*^*Prx1*^; *Pinch2*^−/−^ (dKO), but not single mutant *(Pinch1*^*Prx1*^ or *Pinch2*^−/−^), mice display dwarfism. **a** Representative pictures of 6-week-old male control (*Prx1-Cre*), *Pinch1*^*Prx1*^, *Pinch2*^−/−^, and dKO mice. Scale bar, 1 cm. **b** Growth curve. The results are expressed as the mean ± standard deviation (s.d.). Student’s *t* test, *P < 0.05; **P < 0.01; ***P < 0.001. *N* = 5 control, *Pinch1*^*Prx1*^ and *Pinch2*^−/−^ mice; *N* = 7 dKO mice. **c** Survival curve. *N* = 7 mice per group. Alcian blue-alizarin red double staining of whole-mount skeletons (**d**), rib cages and sterna (**e**), and calvaria (**f**) of P0 control, *Pinch2*^−/−^, *Pinch1*^*Prx1*^, and dKO mice. Scale bar, 5 mm. The red arrow indicates the bifurcation of the sternum in dKO mice. **g** Alcian blue/hematoxylin/Orange G staining of humeral sections from E18.5 and P0 control and dKO mice. Scale bar, 200 mm. **h** Hematoxylin and eosin (H/E) staining of humeral sections from E18.5 and P0 control and dKO mice. Scale bar, 100 μm. **i**–**k** Alcian blue-alizarin red staining of tibial sections from P17 and 6-week-old male control, *Pinch2*^−/−^, *Pinch1*^*Prx1*^, and dKO mice (**i**). Quantification of the lengths of the proliferative zone (PZ) (**j**) and hypertrophic zone (HZ) (**k**). *N* = 5 mice per group. Student’s *t* test. The results are expressed as the mean ± s.d. ***P < 0.001. Scale bar, 400 μm (top and middle) or 80 μm (bottom)

To investigate the role of Pinch1/2 in skeletogenesis, we deleted Pinch1 expression in limb MSCs using *Prx1-Cre* transgenic mice and generated mice with Pinch2 global deletion (*Pinch1*^*Prx1*^*; Pinch2*^−/−^ mice, referred to as dKO mice hereafter). The *Prx1-Cre*, *Pinch1*^*Prx1*^, *Pinch2*^−/−^, and dKO mice were all viable at birth and born at the expected Mendelian ratio. Body size, weight, and length were not significantly different among *Prx1-Cre*, *Pinch1*^*Prx1*^, and *Pinch2*^−/−^ mice (Fig. 1a–c). In contrast, dKO mice developed growth retardation after birth, exhibiting lower body weights than *Prx1-Cre*, *Pinch1*^*Prx1*^, or *Pinch2*^−/−^ mice (Fig. 1a, b). The majority of dKO mice died before 10 weeks of age of undefined cause(s) (Fig. 1c). Alcian blue and alizarin red double staining of P0 skeletons revealed that the dKO mice had markedly smaller skeletons than *Prx1-Cre*, *Pinch1*^*Prx1*^, and *Pinch2*^−/−^ mice (Fig. 1d) and exhibited bifurcation of the sternum (Fig. 1e). At P0, dKO mice displayed a larger unmineralized fontanel than control mice (Fig. 1f), suggesting that intramembranous ossification was also affected in dKO mice. Alcian blue/hematoxylin/Orange G staining of humeral sections from E18.5 and P0 mice showed that while the primary ossification center (POC) was formed in both the control and dKO mice, the length of POC was markedly shorter in dKO mice than in control mice (Fig. 1g). Hematoxylin and eosin (H/E) staining of humeral sections from E18.5 and P0 control and dKO mice showed that cellularity was markedly lower in the dKO growth plate than in the control growth plate (Fig. 1h). Furthermore, Pinch loss disrupted chondrocyte column formation at E18.5 and P0 (Fig. 1h), delayed the formation of the secondary ossification center (SOC) in the tibiae (Fig. 1i), and increased the length of the proliferative zone (PZ) without markedly affecting the length of the hypertrophic zone (HZ) in the tibial growth plate (Fig. 1j, k).

**Fig. 2:**
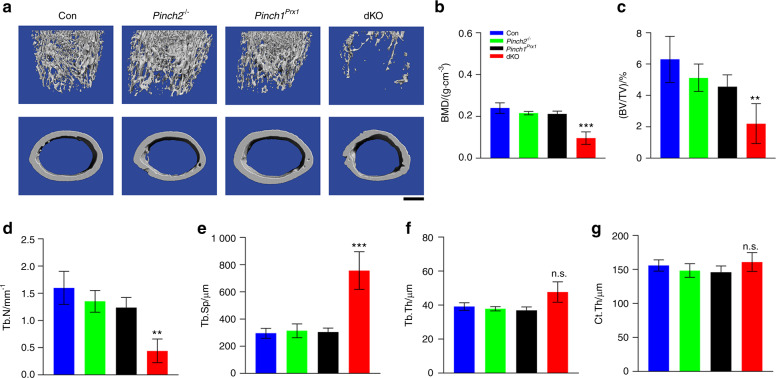
dKO, but not single mutant, mice display severely low bone mass. **a** Three-dimensional (3D) reconstruction from microcomputed tomography (μCT) scans of the distal femurs of 6-week-old male control, *Pinch2*^−/−^, *Pinch1*^*Prx1*^, and dKO mice. Scale bar, 500 μm. Quantitative analyses of bone mineral density (BMD) (**b**), bone volume/tissue volume (BV/TV) (**c**), trabecular number (Tb.N) (**d**), trabecular separation (Tb.Sp) (**e**), trabecular thickness (Tb.Th) (**f**), and cortical thickness (Ct.Th) (**g**). *N* = 5 control, *Pinch1*^*Prx1*^, and *Pinch2*^−/−^ mice; *N* = 7 dKO mice. **P < 0.01; ***P < 0.001 versus control mice, Student’s *t* test. The results are expressed as the mean ± s.d.

Microcomputed tomography (μCT) analysis of the distal femurs of 6-week-old male mice revealed that the bone mineral density (BMD) and bone volume fraction (BV/TV) of dKO mice were dramatically lower than those of age- and sex-matched *Prx1-Cre* and single mutant (*Pinch1*^*Prx1*^ and *Pinch2*^−/−^) mice (Fig. 2a–c). The trabecular number (Tb.N) was higher (Fig. 2d), while the trabecular separation (Tb.Sp) was lower (Fig. 2e) in dKO mice than in control mice. The trabecular thickness (Tb.Th) and cortical thickness (Cort.Th) were slightly higher in the dKO mice than in the control mice, but the differences did not achieve statistical significance (Fig. 2f, g). Female dKO mice displayed lower body weights and body lengths and less severe osteopenia than sex-matched control mice (Supplementary Fig. 1a–e).

### Pinch loss reduces chondrocyte proliferation and cellularity and increases hypertrophic chondrocyte apoptosis, resulting in shortened and broadened limbs

**Fig. 3:**
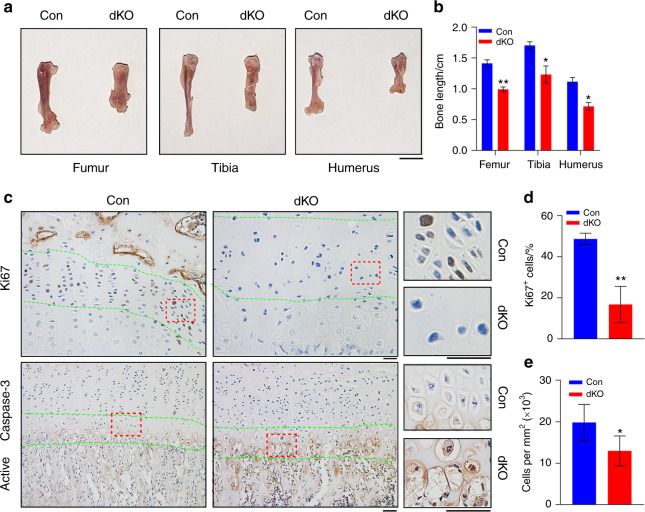
Pinch ablation reduces PZ chondrocyte proliferation and increases HZ chondrocyte apoptosis, resulting in limb shortening. **a** Representative pictures of the femurs, tibiae, and humeri of 6-week-old male control and dKO mice. Scale bar, 5 mm. **b** Quantification of long bone lengths. Student’s *t* test. The results are expressed as the mean ± s.d. *P < 0.05, **P < 0.01, versus control mice. *N* = 8 control mice; *N* = 6 dKO mice. **c**–**e** Immunohistochemical (IHC) staining of tibial sections from 6-week-old male control and dKO mice with an antibody against Ki67 (**c**, top) or active caspase-3 (**c**, bottom). Scale bar, 50 μm. Quantification of Ki67^+^ cells in the PZ (**d**) and cellularity in the PZ (**e**). Quantitative data were obtained from the areas between the two green dashed lines. *N* = 3 mice per group (**d**) or 5 mice per group (**e**). The data are expressed as the mean ± s.d. *P < 0.05, **P < 0.01 versus control mice, Student’s *t* test

Because neither *Pinch1*^*Prx1*^ nor *Pinch2*^−/−^ mice displayed marked skeletal phenotypes, we next focused our investigation on analyzing the phenotypes of dKO mice using *Prx1-Cre* mice as controls. dKO mice displayed dramatically shorter and broader limbs than *Prx1-Cre* mice (Fig. 3a, b). IHC staining of tibial sections using an antibody against Ki67, a specific nuclear marker of cell proliferation, showed a drastic reduction in Ki67-positive chondrocytes in dKO mice compared to control mice (Fig. 3c, d). The expression of active caspase-3, an indicator of apoptosis, was markedly higher in HZ chondrocytes in dKO mice than in control mice (Fig. 3c). Pinch loss decreased the cellularity of the PZ in the tibial growth plate (Fig. 3e).

### Pinch loss downregulates Smad2/3 in PZ chondrocytes and upregulates Runx2 and Col10a1 in PZ and HZ chondrocytes

**Fig. 4:**
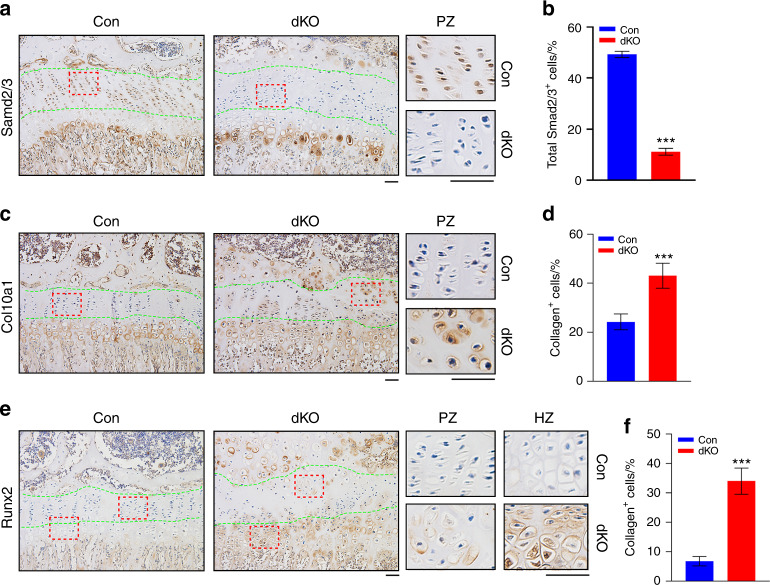
Pinch deletion decreases Smad2/3 expression in PZ chondrocytes but increases the expression of Col10a1 and Runx2 in PZ and HZ chondrocytes. **a**–**f** IHC staining of tibial sections from 6-week-old male control and dKO mice with antibodies against Smad2/3 (**a**), Col10a1 (**c**), and Runx2 (**e**). Scale bar, 50 μm. Quantification of Smad2/3^+^, Col10a1^+^, and Runx2^+^ cells (**b**, **d**, **f**). Quantitative data were obtained from the areas between the two green dashed lines. *N* = 4 mice per group for Smad2/3 (**b**); *N* = 5 mice per group for Col10a1 and Runx2 (**d**, **f**). The data are expressed as the mean ± s.d. ***P < 0.001, versus controls, Student’s *t* test. The number of cells in the areas between the dotted lines was determined

We performed IHC staining of tibial sections from mice of the two genotypes and found that the protein expression of Smad2/3 was dramatically lower in PZ chondrocytes in dKO mice than in control mice (Fig. 4a, b). The reduction in Smad2/3 expression in dKO mice was specific to PZ chondrocytes, as the expression of Smad2/3 in HZ chondrocytes and subchondral bone was not lower in dKO mice than in control mice.

Col10a1 is normally expressed at very low levels in PZ chondrocytes, while its expression is relatively higher in HZ chondrocytes. Consistently, we found that Col10a1 was barely detectable in PZ chondrocytes and strongly detected in HZ chondrocytes in the tibial growth plate of control mice (Fig. 4c, d). Strikingly, Col10a1 was expressed at a high level in the PZ chondrocytes of dKO mice (Fig. 4c, d). Runx2 is a direct upstream transcriptional activator of *Col10a1*,^21^ and chondrocyte hypertrophy is mainly regulated by Runx2.^52^ We found that Runx2 was dramatically upregulated in PZ chondrocytes in the tibial growth plates of dKO mice compared to those of control mice (Fig. 4e, f). A number of Col10a1- and Runx2-expressing hypertrophic chondrocytes were observed in the growth plates of dKO mice close to the SOC (Fig. 4c, e). Notably, the expression of Runx2 was also higher in HZ chondrocytes in the tibial growth plates of dKO mice than in the tibial growth plates of control mice (Fig. 4e).

### Pinch1 and Smad2/3 interact with each other and colocalize in the nuclei of ATDC5 cells

**Fig. 5:**
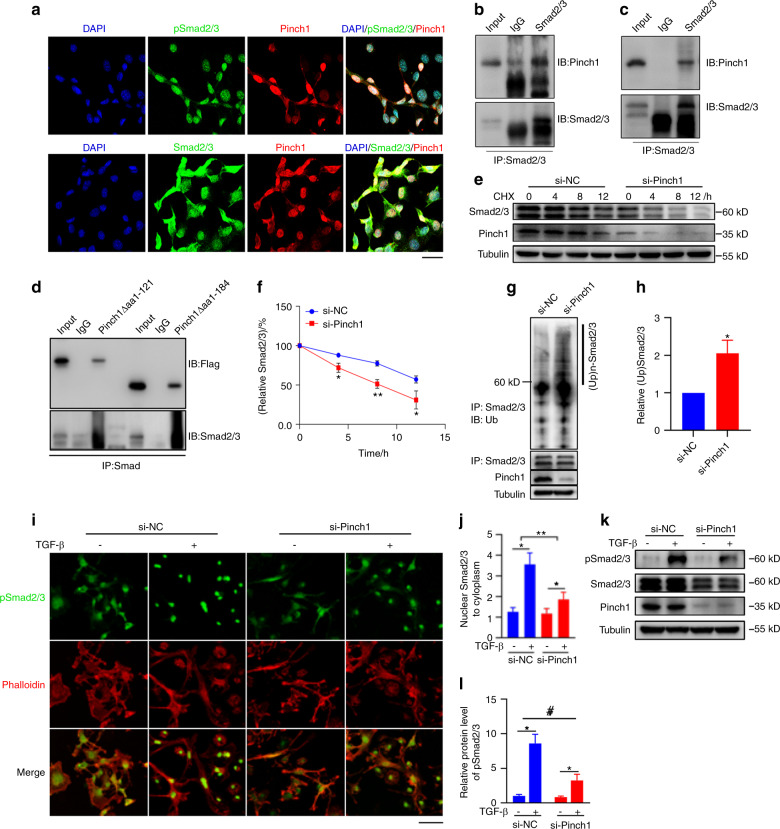
Pinch loss impairs TGF-β1/Smad2/3 signaling. **a** Immunofluorescence (IF) staining. ATDC5 cells (2 × 10^4^ cells/well) were seeded in confocal dishes (SPL Life Science) for 24 h, and then subjected to IF staining using the indicated antibodies. Scale bar, 20 µm. **b** Immunoprecipitation (IP) assay. COS-7 cells (2 × 10^6^ cells/well) were seeded in 100-mm dishes. Twenty-four hours later, the cells were transfected with 5 μg Pinch1 expression plasmids. After 24 h, whole-cell extracts were prepared, immunoprecipitated with a Smad2/3 antibody, and subjected to western blot analysis using a Pinch1 (top) or Smad2/3 (bottom) antibody. **c** IP assay. Whole-cell extracts from ATDC5 chondrocyte-like cells were immunoprecipitated with a Smad2/3 antibody and then subjected to western blot analysis using a Pinch1 (top) or Smad2/3 (bottom) antibody. **d** IP assay. COS-7 cells were transfected with the indicated Flag-Pinch1 deletion mutant expression vector. After 36 h, whole-cell protein extracts were immunoprecipitated with an M2 antibody and then subjected to western blot analysis using an M2 (top) or Smad2/3 antibody (bottom). **e**, **f** Cycloheximide (CHX) experiment. Primary Pinch2 KO BMSCs were transfected with control or Pinch1 siRNA. Twenty-four hours later, the cells were treated with 10 μg·mL^−1^ CHX. Cell lysates were subjected to western blot analysis of Smad2/3 expression. Quantitative analysis of Smad2/3 expression, normalized to β‐actin, from three independent experiments (**f**). **g**, **h** Smad2/3 ubiquitination. Primary BMSCs from Pinch2 KO mice were transfected with control (si-NC) or Pinch1 siRNA (si-Pinch1). Twenty-four hours later, whole‐cell extracts were immunoprecipitated with an anti‐Smad2/3 antibody, and then subjected to western blot analysis of ubiquitin (Ub) (**g**). Quantitative analysis of (Ub)n‐Smad2/3 from three independent experiments (**h**). *P < 0.01 versus si-NC. **i**, **j** IF staining. Primary BMSCs from Pinch2 KO mice were transfected with si-NC or si-Pinch1. Twenty-four hours later, the cells were treated with or without 10 ng·mL^−1^ TGF-β1 for 30 min and then subjected to IF staining using a Smad2/3 antibody and phalloidin. Scale bar, 20 µm. Quantitative analysis of pSmad2/3 expression normalized to tubulin, from three independent experiments (**j**). **k**, **l** Western blotting. Primary BMSCs from Pinch2 KO mice were transfected with si-NC or si-Pinch1. Twenty-four hours later, the cells were treated with 10 ng·mL^−1^ TGF-β1 for 30 min and then subjected to western blotting. Quantitative analysis of pSmad2/3 expression normalized to tubulin, from three independent experiments (**l**). *P < 0.01 versus si-NC

We performed confocal microscopy analysis and found that Pinch1 and Smad2/3 colocalized in the nuclei of ATDC5 chondrocyte-like cells (Fig. 5a). Immunoprecipitation (IP) assays using whole-cell extracts from COS-7 cells overexpressing Pinch1 (Fig. 5b) or from ATDC5 cells (Fig. 5c) revealed that Pinch1 interacted with Smad2/3 in both cell types. Deletion of the aa 1–121 region, which contains LIMS domains 1 and 2, or the aa 1–184 region, which contains LIMS domains 1–3, from Pinch1 did not abolish its interaction with Smad2/3 (Fig. 5d), suggesting that the C-terminal region of Pinch1, which is composed primarily of LIM domains 4 and 5, mediates the interaction between the two factors.

### Pinch1 loss increases Smad2/3 ubiquitination and degradation and decreases TGF-β-induced Smad2/3 phosphorylation and nuclear localization

We performed cycloheximide experiments in primary bone marrow stromal cells (BMSCs) from Pinch2 KO mice with or without Pinch1 siRNA knockdown. The results showed that Pinch loss accelerated the degradation of Smad2/3 proteins in primary BMSCs (Fig. 5e, f). Pinch loss in these cells increased the level of Smad2/3 ubiquitination (Fig. 5g, h). Immunofluorescence (IF) staining of primary BMSCs showed that TGF-β1 rapidly and dramatically increased Smad2/3 nuclear localization (Fig. 5i, j), which was markedly decreased by Pinch loss (Fig. 5i, j).

Finally, Pinch loss significantly reduced TGF-β-induced Smad2/3 phosphorylation in BMSCs (Fig. 5k, l).

### Pinch loss upregulates sclerostin in HZ chondrocytes and reduces bone formation

**Fig. 6:**
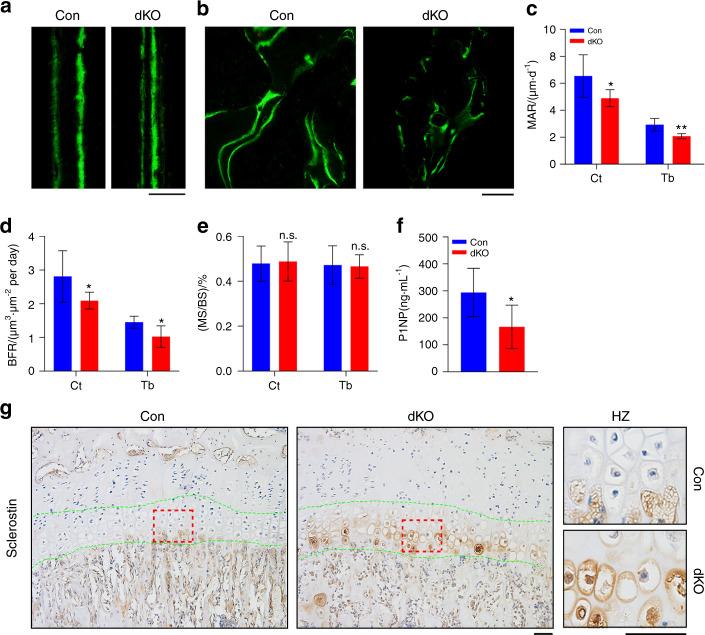
Pinch loss increases sclerostin expression in HZ chondrocytes and reduces bone formation. **a**–**e** Calcein double labeling. Images of calcein double labeling of the femoral diaphyseal cortical bones (**a**) and metaphyseal cancellous bones (**b**) of 6-week-old male control and dKO mice. Scale bar, 50 μm. Quantification of MAR (**c**), MS/BS (**d**), and BFR (**e**) of the femoral diaphyseal cortical bones (Ct) and metaphyseal cancellous bones (Tb) of 6-week-old male control and dKO mice. *N* = 7 mice for control cortical bone MAR; *N* = 6 mice for dKO cortical bone MAR; *N* = 6 mice for both control and dKO trabecular bone MAR; *N* = 8 mice for control cortical bone BFR; *N* = 4 mice for dKO cortical bone BFR; *N* = 5 mice for control trabecular bone BFR; *N* = 8 mice for dKO trabecular bone BFR; *N* = 8 mice for control trabecular and cortical bone MS/BS; and *N* = 6 mice for dKO trabecular and cortical bone MS/BS. The data are expressed as the mean ± s.d. Student’s *t* test. *P < 0.05; **P < 0.01. **f** ELISA of serum levels of procollagen type 1 amino-terminal propeptide (P1NP). *N* = 5 control mice; *N* = 8 dKO mice. Student’s *t* test. The results are expressed as the mean ± s.d. *P < 0.05. **g** IHC staining of tibial sections from 6-week-old male control and dKO mice with an antibody against sclerostin. Scale bar, 50 μm

We explored the potential mechanism(s) through which Pinch loss causes osteopenia in dKO. The results of the calcein double labeling experiments revealed that the mineralization apposition rate (MAR) and bone formation rate (BFR) of the femoral diaphyseal cortical bones and metaphyseal cancellous bones were significantly lower in dKO mice than in control mice (Fig. 6a–d). Pinch loss did not alter the mineralizing surface per bone surface (MS/BS) (Fig. 6e). Consistent with the reductions in the MAR and BFR, the level of serum procollagen type 1 amino-terminal propeptide (P1NP), a bona fide bone formation marker, was significantly lower in 6-week-old male dKO mice than in control mice (Fig. 6f). Sclerostin is a secreted inhibitor of Wnt/β-catenin signaling and bone formation.^53–55^ We found that its expression was upregulated in the HZ chondrocytes of dKO mice compared to those of control mice (Fig. 6g).

### Pinch loss decreases osteoblast formation from bone marrow cells

**Fig. 7:**
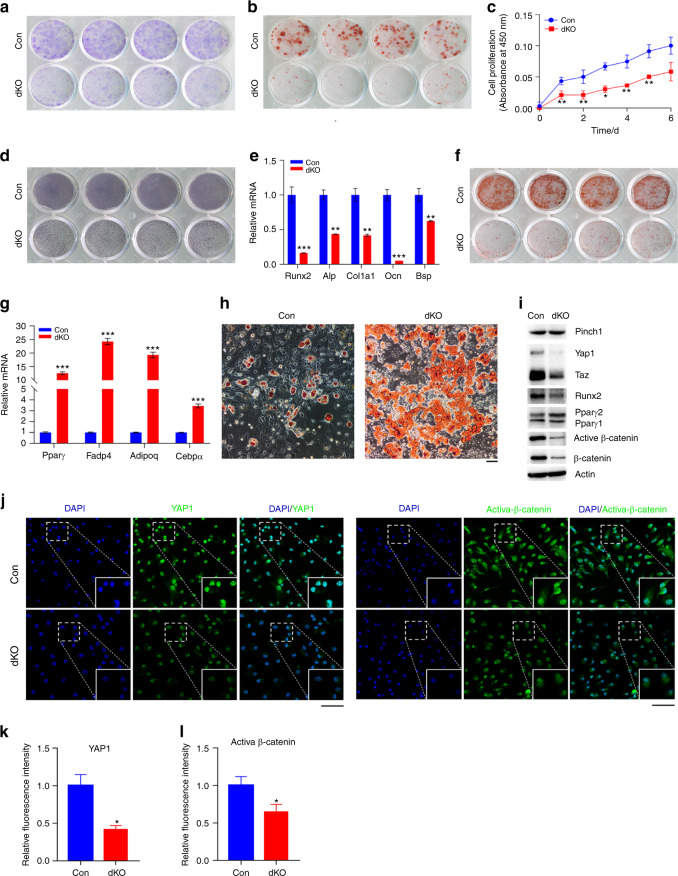
Pinch loss inhibits the osteoblastic differentiation capacity but enhances the adipogenic differentiation capacity of BMSCs. **a** The colony forming unit-fibroblast (CFU-F) assay. Nucleated bone marrow cells from 6-week-old male control and dKO mice were seeded in a six-well plate at a cell density of 2 × 10^6^ per well, cultured using the Mouse MesenCult Proliferation Kit (CFU-F assay) for 14 days and subjected to Giemsa staining. **b** The colony forming unit-osteoblast (CFU-OB) assay. Nucleated bone marrow cells from 5-week-old male control and dKO mice were seeded in a six-well plate at a density of 4 × 10^6^ per well, cultured in osteoblast differentiation media for 21 days (the medium was changed every 48 h) and subjected to alizarin red staining. **c** The BMSC proliferation assay. BMSCs were seeded in a 96-well plate at a density of 2 000 cells per well. The absorbance was measured at 0, 2, 4, and 6 days. *P < 0.05; **P < 0.01 versus control mice, Student’s *t* test. The results are expressed as the mean ± s.d. **d**, **e** Primary BMSCs from control and dKO mice were cultured with osteoblast differentiation medium for 7 days and then subjected to ALP staining (**d**) or quantitative real-time PCR (qRT-PCR) to evaluate the expression of the indicated genes, which was normalized to the level of *Gapdh* mRNA (**e**). *N* = 3 mice per group. **f** Alizarin red S staining. BMSCs were cultured in osteoblast differentiation medium for 7 days and then in mineralization-inducing medium for 7 days. **g**, **h** Adipogenic differentiation. BMSCs were cultured in adipogenic differentiation medium from the MesenCult™ Adipogenic Differentiation Kit for 9 days and then subjected to qPCR to evaluate the expression of the indicated genes, which was normalized to the level of *Gapdh* mRNA (**g**) or subjected to Oil Red O staining (**h**). *N* = 3 mice per group. Scale bar, 100 μm. **i** Western blotting. Protein extracts isolated from BMSCs from 6-week-old male control and dKO mice of the two genotypes were subjected to western blotting. **j**–**l** IF staining. BMSCs from 6-week-old male control and dKO mice of the two genotypes were subjected to IF staining using the indicated antibodies or DAPI. Scale bar, 25 µm. Quantification of Yap1 (**k**) and β-catenin (**l**) expression

To determine whether Pinch loss in limb MSCs impacts bone marrow cells, we performed a colony forming unit-fibroblast (CFU-F) assay using primary bone marrow cells from mice of the two genotypes. The results showed that while CFU-F colonies were formed in both groups, the CFU-F colonies were strikingly smaller in dKO cultures than in control cultures (Fig. 7a). Furthermore, Pinch loss reduced the formation of colony forming unit-osteoblasts (CFU-OBs) (i.e., osteoprogenitors) in the bone marrow cell cultures (Fig. 7b), and dKO BMSCs displayed defective proliferation (Fig. 7c).

### Primary BMSCs from dKO mice display decreased osteoblastic but increased adipogenic differentiation capacity

We next determined whether Pinch loss in Prx1-expressing cells affects BMSC differentiation potential. Primary BMSCs were isolated from control and dKO mice and induced to differentiate into osteoblasts or adipocytes as described in the “Methods” section. Osteoblasts derived from dKO BMSCs displayed dramatically lower alkaline phosphatase (Alp) protein expression and *Runx2*, *Alp, Col1a1*, osteocalcin, and bone sialoprotein mRNA expression than those derived from control BMSCs (Fig. 6d, e). Alizarin red staining revealed lower calcium deposition in dKO cultures than in control cultures (Fig. 6f). In contrast, dKO BMSCs displayed higher expression of adipocyte genes, including those encoding peroxisome proliferator-activated receptor gamma (Ppar-γ), a major regulator of adipogenic differentiation, fatty acid binding protein 4, adiponectin (Adipoq), and CCAAT-enhancer binding protein α, than control BMSCs (Fig. 7g). Adipogenesis was markedly enhanced in dKO cultures relative to control cultures, as measured by Oil Red O staining (Fig. 7h).

### Primary BMSCs from dKO mice express reduced proteins levels of β-catenin, Yap1/Taz, and Runx2 and increased protein levels of Ppar-γ

Western blotting revealed lower protein levels of active and total β-catenin and Yap1/Taz in dKO BMSCs than in control BMSCs (Fig. 7i). Furthermore, Pinch loss decreased the protein level of the key osteoblast transcription factor Runx2 but increased the level of Pparγ in primary BMSC cultures (Fig. 7i). IF staining showed that β-catenin and Yap1/Taz proteins were detected at high levels in the nuclei of control BMSCs but that their levels were dramatically lower in dKO BMSCs (Fig. 7j–l). It should be noted that the expression of Pinch1 protein was not lower in dKO BMSCs than in control BMSCs (Fig. 7i).

### Pinch loss in limb MSCs increases the expression of Rankl in HZ chondrocytes and promotes osteoclast formation and bone resorption

**Fig. 8:**
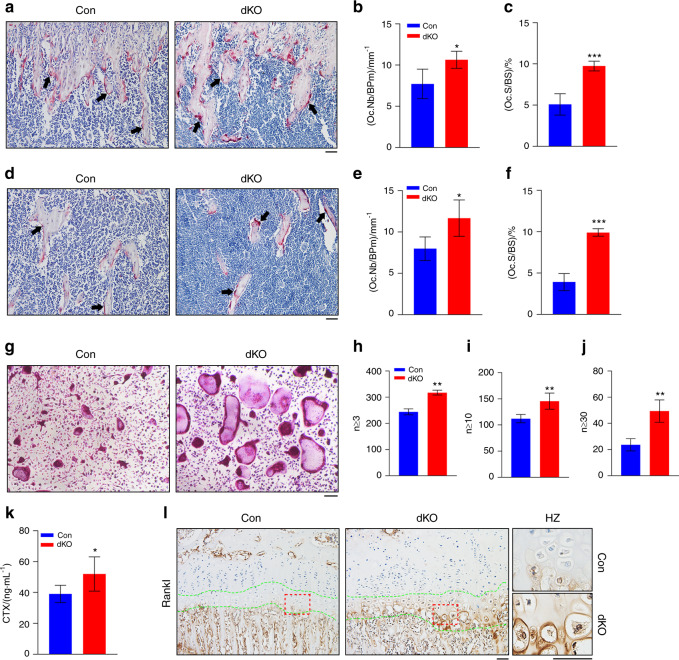
Pinch loss upregulates Rankl in HZ chondrocytes and promotes osteoclast formation in vitro and in bone. **a**–**f** Tartrate-resistant acid phosphatase (TRAP) staining. Tibial sections from 6-week-old male control and dKO mice were used for TRAP staining (**a**, **d**). The osteoclast surface/bone surface (Oc.S/BS) (**c**, **f**) and osteoclast number/bone perimeter (Oc.N/BPm) (**b**, **e**) of the primary and secondary spongiosa bones from mice of the two genotypes were measured using Image-Pro Plus 7.0 (**c**–**g**). The arrow indicates osteoclasts located on the trabecular bone surface. Scale bar, 50 μm. *P < 0.05, ***P < 0.001. *N* = 5 mice per group. Student’s *t* test. The results are expressed as the mean ± s.d. **g**–**j** In vitro osteoclast formation. Primary BMMs from 6-week-old control and dKO male mice were first cultured in proliferation medium (α-MEM containing 10% FBS and 10 ng·mL^−1^ human recombinant M-CSF) for 3 days followed by differentiation medium (proliferation medium plus 50 ng·mL^−1^ human recombinant RANKL) for 4–9 days and then subjected to TRAP staining (**g**). TRAP^+^ MNCs (≥3, 10, or 30 nuclei) per well were scored (**h**–**j**). The data are expressed as the mean ± s.d. Student’s *t* test. **P < 0.01. Scale bar, 100 μm. **k** ELISA of serum levels of collagen type I cross-linked C-telopeptide (CTX1). Sera were collected from 6-week-old male control and dKO mice and subjected to ELISA for CTX1. *N* = 5 control mice; *N* = 6 dKO mice. Student’s *t* test. The results are expressed as the mean ± s.d. **l** IHC staining of tibial sections from 6-week-old male control and dKO mice with an anti-Rankl antibody. Scale bar, 50 μm

We next investigated whether Pinch loss in limb MSCs impacts osteoclast formation and bone resorption. Staining of tibial sections for the osteoclast enzyme tartrate-resistant acid phosphatase (TRAP) revealed that osteoclast formation was higher in bone from dKO mice than in bone from control mice (Fig. 8a). Specifically, the osteoclast number/bone perimeter (Oc.Nb/BPm) and osteoclast surface/bone surface (Oc.S/BS) were significantly higher in both primary and secondary spongiosa in the bones of dKO mice than in the bones of control mice (Fig. 8b–f). Osteoclast formation was also dramatically increased in primary bone marrow monocyte (BMM) cultures from dKO mice compared to BMM cultures from control mice (Fig. 8g). The numbers of TRAP^+^ multinucleated cells with more than 3, 10, or 30 nuclei were all significantly increased in dKO BMM cultures relative to those in control BMM cultures (Fig. 8h–j). Consistent with the increase in osteoclast formation, the serum level of collagen type I cross-linked C-telopeptide 1 (CTX1), an indicator of osteoclastic bone resorption, was significantly higher in dKO mice than in control mice (Fig. 8k). Hypertrophic chondrocytes are known to express Rankl.^56^ We determined whether Pinch loss affects Rankl expression and found that its expression was higher in the HZ chondrocytes of dKO mice than in the HZ chondrocytes of control mice in the tibial growth plate (Fig. 8i).

### Deleting Pinch1 in chondrocytes and Pinch2 globally in mice results in severe osteopenia with subtle limb shortening

**Fig. 9:**
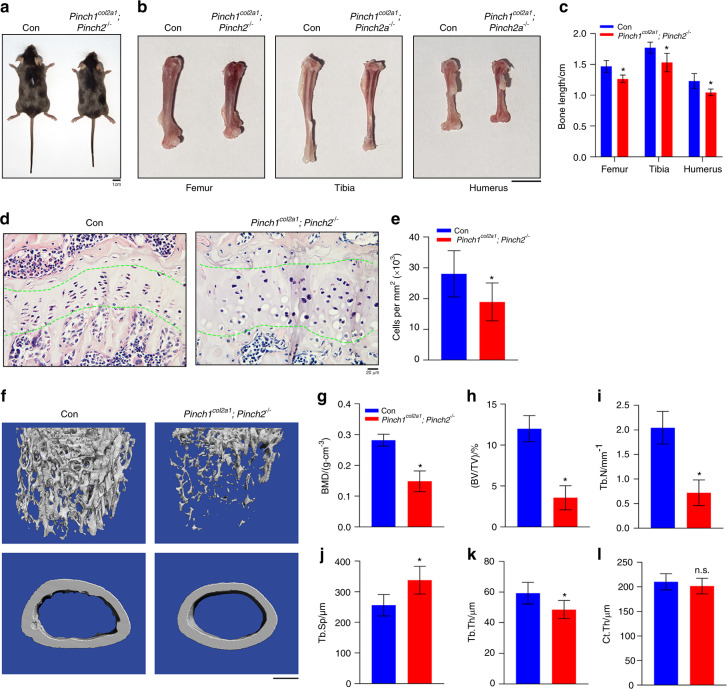
Deleting Pinch1 in chondrocytes and global Pinch2 deletion causes subtle limb shortening and low bone mass in mice. **a** Whole-body photographs of 2-month-old control and *Pinch1*^*Col2a1*^*; Pinch2*^−/−^ mice. *N* = 5 mice per group. Scale bar, 1 cm. **b**, **c** The femurs, tibiae and humeri of 3-momth-old male control and *Pinch1*^*Col2a1*^*; Pinch2*^−/−^ mice (**b**). Quantitative data of long bones length (**c**). *P < 0.05. *N* = 5 mice per group. Scale bar, 5 mm. **d**, **e** H/E staining of tibial sections from 3-month-old male control and *Pinch1*^*Col2a1*^*; Pinch2*^−/−^ mice. Quantification of the data in **d** (**e**). The quantitative data were obtained from the areas between the two green dashed lines. *N* = 5 mice per group. Student’s *t* test. The results are expressed as the mean ± s.d. *P < 0.05. Scale bar, 20 μm. **f** 3D reconstruction from μCT scans of the femurs of 3-month-old male control and *Pinch1*^*Col2a1*^*; Pinch2*^−/−^ mice. Scale bar, 500 μm. **g**–**l** Quantitative analysis of the BMD, BV/TV, Tb.N, Tb.Sp, Tb.Th, and Ct.Th. *N* = 5 mice per group. Student’s *t* test. The results are expressed as the mean ± s.d. *P < 0.05

To determine whether Pinch1/2 play direct roles in chondrocytes and skeletogenesis, we deleted Pinch1 expression in chondrocytes using *Col2a1-Cre* transgenic mice and generated mice with global Pinch2 deletion (*Pinch1*^*Col2a1*^*; Pinch2*^−/−^ mice). The breeding strategy used to generate these mice is described in detail in the “Methods” section. *Col2a1-Cre* mice were used as controls. *Pinch1*^*Col2a1*^*; Pinch2*^−/−^ mice displayed lower body weights and body lengths than age- and sex-matched *Col2a1-Cre*, *Pinch1*^*Col2a1*^ and *Pinch2*^−/−^ mice (Supplementary Fig. 2a–d). Compared to control mice, two-month-old *Pinch1*^*Col2a1*^*; Pinch2*^−/−^ mice displayed slightly reduced body weights with subtle limb shortening (Fig. 9a–c). Similar to *Pinch1*^*Prx1*^*; Pinch2*^−/−^ mice, *Pinch1*^*Col2a1*^*; Pinch2*^−/−^ mice displayed disrupted chondrocyte column formation and reduced cellularity in the growth plate (Fig. 9d, e). μCT analysis of the femurs of 3-month-old male *Pinch1*^*Col2a1*^*; Pinch2*^−/−^ mice revealed more severe osteopenia than that in the femurs of control mice (Fig. 9f). Specifically, the BMD, BV/TV and Tb.N were lower, and the Tb.Sp was higher in *Pinch1*^*Col2a1*^*; Pinch2*^−/−^ mice than in control mice (Fig. 9g–j). The Tb.Th was slightly but significantly lower in *Pinch1*^*Col2a1*^*; Pinch2*^−/−^ mice than in control mice (Fig. 9k). The Cort.Th was not significantly different between *Pinch1*^*Col2a1*^*; Pinch2*^−/−^ mice and control mice (Fig. 9j). Unlike *Pinch1*^*Prx1*^*; Pinch2*^−/−^ mice, *Pinch1*^*Col2a1*^*; Pinch2*^−/−^ mice did not die prematurely and displayed a normal lifespan compared to that of control mice.

## Discussion

This study demonstrates critical roles for Pinch1/2 in the regulation of skeletogenesis through the control of endochondral ossification. We established that Pinch1/2 regulate chondrocyte function at least in part by modulating TGF-β1 signaling in chondrocytes and their precursors. These findings will improve our understanding of endochondral ossification, abnormalities of which cause dwarfism and low bone mass, which both have broad clinical significance.

Our results demonstrate that Pinch1/2 regulate multiple aspects of chondrocytes, including cell proliferation, differentiation, hypertrophy, and apoptosis. Pinch loss in limb MSCs largely reduces chondrocyte proliferation, as demonstrated by a dramatic decrease in the number of Ki67^+^ cells in the PZ of the long bone growth plates. Pinch loss accelerates chondrocyte differentiation and hypertrophy. Strikingly, the majority of the PZ chondrocytes of dKO mice express a high level of Col10a1, a marker of hypertrophic chondrocytes. The chondrocyte column, an important feature of the growth plate, is largely disrupted by Pinch deficiency, and Pinch loss delays SOC formation. Together, these cellular defects largely impair the development of the long bone growth plates, leading to severe limb shortening in dKO mice.

The results of the present study suggest that Pinch1/2 regulate skeletogenesis at least in party by modulating TGF-β/Smad2/3 signaling in chondrocytes and their precursors. This notion is supported by the following lines of evidence: (1) lower expression of Smad2/3 in PZ chondrocytes in the long bone growth plates of dKO mice than those of control mice in vivo; (2) reduced TGF-β-induced Smad2/3 phosphorylation in Pinch1/2-deficient BMSCs compared to control BMSCs in vitro; (3) colocalization of Pinch1 and Smad2/3 in the nuclei of chondrocytes; (4) the interaction of Pinch1 with Smad2/3 through the C-terminal region of Pinch1; (5) increase in Smad2/3 protein ubiquitination and degradation due to Pinch1 loss; and (6) the well-established roles of TGF-β/Smad2/3 signaling in the regulation of chondrocyte function and skeletogenesis.^24–30^ Thus, we demonstrate a novel mechanism through which Pinch1/2 modulates TGF-β/Smad2/3 signaling during skeletogenesis. The molecular mechanism(s) through which Pinch1/2 stabilize Smad2/3 remain to be determined.

Runx2 upregulation induced by Pinch loss may play an important role in promoting chondrocyte differentiation and hypertrophy in dKO mice because chondrocyte differentiation and hypertrophy are mainly regulated by Runx2.^52^ TGF-β represses chondrocyte differentiation and hypertrophy,^31,32^ and TGF-β signaling suppresses Runx2 function through Smad3.^33,57,58^ Thus, it is likely that downregulation of Smad2/3 induced by Pinch loss leads to upregulation of Runx2, which promotes chondrocyte differentiation and hypertrophy.

It is known that hypertrophic chondrocytes express both sclerostin and Rankl.^53,56,59^ The results of the present study show that HZ chondrocytes in the growth plates of dKO mice express abundant sclerostin and Rankl. At increased levels, both factors could diffuse into the bone marrow cavity and impact osteoblast and osteoclast formation and differentiation, respectively, leading to low bone mass in mutant mice. The molecular mechanism(s) through which Pinch loss in limb MSCs increases osteoclast formation remain to be determined in future studies.

We found that primary cultured BMSCs from dKO mice displayed lower osteoblastic but higher adipogenic and differentiation capacity than primary cultured BMSCs from control mice. However, our western blotting results show that the expression of Pinch1 is not decreased in dKO BMSCs. This result suggests that (1) dKO BMSCs are not derived from the Prx1-expressing limb MSC population and that (2) dKO BMSCs are influenced in the bone microenvironment by signal(s) induced by Pinch loss in limb MSCs. Increased sclerostin expression by dKO HZ chondrocytes may play a role in this regulation. We recently demonstrated that Yap1/Taz play an important role in the control of MSC differentiation fate by promoting osteoblastic differentiation but inhibiting adipogenic differentiation of MSCs.^60^ The results of the present study reveal that dKO BMSCs express dramatically lower levels of Yap1/Taz proteins than control BMSCs, which may contribute in part to the altered BMSC differentiation capacity of dKO BMSCs. However, the molecular mechanism(s) through which Pinch loss in limb MSCs downregulates Yap1/Taz in BMSCs remain to be determined.

It is interesting to compare the skeletal phenotypes of the Pinch1/2 dKO mice (used in this study) and *Kindlin-2*^*Prx1*^ mice (used in our previous study^61^). Deletion of Kindlin-2 or Pinch1/2 in Prx1-expressing cells causes severe limb shortening. Furthermore, *Kindlin-2*^*Prx1*^ mice display complete loss of the skull; this striking phenotype is not observed in Pinch1/2 dKO mice. Nonetheless, P0 dKO mice did display a larger unmineralized posterior fontanel than control mice (Fig. 1f), suggesting that intramembranous ossification is also affected by Pinch loss.

In this study, we demonstrate a functional redundancy of Pinch1 and Pinch2 in the control of skeletogenesis. Interestingly, a similar functional redundancy of both factors was observed in the heart under pathological, but not physiological, conditions.^62^

Based on the findings of this study and those of other studies, we propose a working model to explain how Pinch controls chondrogenesis and bone mass. Pinch regulates TGF-β/Smad2/3 signaling to maintain Runx2 at a proper level, which keeps chondrocytes in a proliferative state and prevents them from undergoing premature differentiation, hypertrophy, and apoptosis. In the absence of Pinch, TGF-β/Smad2/3 signaling is impaired due to reduced expression and activation and accelerated degradation of Smad2/3, which reduces chondrocyte proliferation and survival and upregulates Runx2. Upregulation of Runx2 accelerates chondrocyte differentiation and hypertrophy. These alterations impair the development of the growth plates, leading to chondrodysplasia and limb shortening. Furthermore, Pinch loss largely promotes the expression of sclerostin and Rankl in HZ chondrocytes, which reduces bone formation and increases resorption, leading to a low bone mass phenotype. Collectively, Pinch1/2, through being expressed in chondrocytes and their precursors, play a critical role in the control of chondrogenesis and bone mass.

## Methods

### Animal studies

*Prx1-Cre* transgenic mice,^63^ *Col2a1-Cre* mice,^64^ and *Pinch1*^*f/f*^ and *Pinch2*^−/−^ mice^62^ were previously described. To obtain the double mutant mice, *Pinch1*^*f/f*^ mice were first crossed with *Pinch2*^−/−^ mice to generate *Pinch1*^*f/+*^*; Pinch2*^*+/*^^−^ mice. *Pinch1*^*f/+*^*; Pinch2*^*+/−*^ mice were then crossed to each other to generate *Pinch1*^*f/f*^*; Pinch2*^−/−^ mice. Hemizygous *Prx1-Cre* mice were crossed with *Pinch1*^*f/f*^*; Pinch2*^−/−^ mice to generate *Prx1-Cre; Pinch1*^*f/+*^*; Pinch2*^*+/−*^ mice. Finally, *Prx1-Cre; Pinch1*^*f/+*^*; Pinch2*^*+/−*^ mice were bred with *Pinch1*^*f/f*^*; Pinch2*^−/−^ mice to generate *Prx1-Cre; Pinch1*^*f/f*^*; Pinch2*^−/−^ mice (*Pinch1*^*Prx1*^*; Pinch2*^−/−^ or dKO mice) and other genotypes. A similar breeding strategy was used to generate *Col2a1-Cre; Pinch1*^*f/f*^*; Pinch2*^−/−^ (*Pinch1*^*Col2a1*^*; Pinch2*^−/−^) mice. The mice used in this study, including *Prx1-Cre*, *Col2a1-Cre*, *Pinch1*^*f/f*^ and *Pinch2*^−/−^ mice, were maintained in our laboratory and bred with normal C57BL/6 mice for more than ten generations. All animal experiments were conducted in the specific pathogen-free Experimental Animal Center of Southern University of Science and Technology. Animals were housed four/cage at 20 °C–24 °C, exposed to a 12-h/12-h light/dark cycle, and given water and rodent chow ad libitum. The mice were monitored daily. All animal protocols were approved by the Institutional Animal Care and Use Committee at the Southern University of Science and Technology. The age, sex, and number of the mice used for each experiment are specified in the figure legends.

### Histology, histomorphometry, and immunohistochemistry

At the time of euthanasia, bone tissues were dissected, fixed, decalcified, and embedded in paraffin as previously described.^65^ Five-micron sections were used for H/E staining, alcian blue staining, toluidine blue staining, and TRAP staining as previously described.^61,66^ For histomorphometry, parameters such as the Oc.S/BS and Oc.Nb/BPm in both the primary and secondary spongiosa, Ob.S/BS, Ob.Nb/BPm of the metaphyseal cancellous bone, lengths of the SOC, PZ, and HZ, and growth plate cellularity of the tibiae and knee joints were measured using Image-Pro Plus 7.0 software (Media Cybernetics Inc.) as we described.^61,66^ For immunohistochemistry, 5-μm sections were deparaffinized with xylene and rehydrated in a descending series of ethanol. Antigen retrieval was performed using citrate buffer (10 mmol·L^−1^, pH 6.0). Endogenous peroxidase activity was blocked with peroxidase-blocking solution (Dako), and protein was blocked with normal horse serum (Vector). The sections were incubated with primary antibodies in a slide staining tray at 4 °C overnight and then incubated with horse biotinylated anti-mouse/rabbit IgG secondary antibody (Vector) followed by streptavidin-horseradish peroxidase (Vector). Immunoreactivity was visualized by the DAB Peroxidase Substrate Kit (Vector) according to the manufacturer’s instructions.

### Calcein double labeling and MAR, MS/BS, and BFR measurement

Calcein double labeling and MAR, MS/BS, and BFR measurements were performed as previously described.^65^

### μCT

Fixed nondemineralized bones were subjected to μCT analysis at the Department of Biology of Southern University of Science and Technology using a Bruker CT imaging system (SkyScan 1172 Micro-CT, Bruker MicroCT, Kontich, Belgium) following the standards of techniques and terminology recommended by the American Society for Bone and Mineral Research (ASBMR).^67^

### Quantitative real-time PCR (qRT-PCR) and western blot analysis

RNA and protein isolation, qRT-PCR, and western blot analysis were performed as previously described.^68^ The specific primers for gene expression analysis are listed in Supplementary Table 1. Primary antibody information is listed in Supplementary Table 2.

### Alcian blue-alizarin red double staining of the skeleton

Alcian blue-alizarin red double staining of the skeleton was performed as previously described.^61^

### ELISA

Serum levels of P1NP were measured using the RatLaps EIA Kit (Immunodiagnostic Systems Limited, Gaithersburg, MD, USA, cat#: AC-33F1) according to the manufacturer’s instructions. Serum levels of CTX1, a degradation product of type I collagen that forms during osteoclastic bone resorption, were measured using the RatLaps EIA Kit (Immunodiagnostic Systems Limited, Gaithersburg, MD, USA, cat#: AC-06F1) as previously described.^69^

### Primary BMSC culture and CFU-F and CFU-OB assays

Primary BMSCs were isolated from tibiae and femurs as previously described.^65^ The CFU-F assay and CFU-OB assay were performed as previously described.^70^

### In vitro BMSC proliferation and differentiation assays

Mouse primary BMSCs were isolated and cultured as described previously.^70^ To evaluate BMSC proliferation, the number of attached cells was assayed by the Cell Counting Kit-8 (Beyotime) assay according to the manufacturer’s instructions. The optical density at 450 nm was determined with a microplate reader (PerkinElmer). For osteogenic differentiation, BMSCs were cultured in osteogenic medium (α-MEM containing 10% FBS and 50 μg·mL^−1^ ascorbic acid) for 7 days and then stained for ALP using a BCIP/NBT Alp color development kit (Beyotime, China) or cultured in osteogenic medium for 7 days followed by mineralization-inducing medium (osteogenic medium plus 2.5 mmol·L^−1^ β-glycerophosphate for 7 days and then subjected to alizarin red S (40 mmol·L^−1^, pH 4.2) (Sigma) staining. For adipogenic differentiation, BMSCs were cultured with reagents from the MesenCult™ Adipogenic Differentiation Kit (STEMCELL Technologies) for 9 days and then stained with Oil Red O (Sigma).

### In vitro and in vivo osteoclast differentiation

Isolation of nonadherent BMMs and in vitro and in vivo osteoclast assays were conducted as previously described.^69^

### TUNEL staining

Cell survival was evaluated using the ApopTag Peroxidase In Situ Apoptosis Detection Kit according to the manufacturer’s instructions (EMD Millipore Corporation, Temecula, CA, USA, cat#: S7100) as previously described.^61^

### DNA constructs and transfection

To generate pCMV/Flag-Pinch1 expression plasmids expressing full-length and truncated forms of Pinch1, DNA elements encoding full-length and respective Pinch1 regions (aa 1–121, aa 1–184) obtained by PCR were subcloned into the HindIII/XhoI sites of the pcDNA3.1(+)-3FLAG vector. All sequences were verified by automatic DNA sequencing.

### IP assay

Whole-cell extracts (1 000 μg) isolated from ATDC5 chondrocyte-like cells or COS-7 cells overexpressing Pinch1 were incubated with 3 μg Smad2/3 antibody overnight at 4 °C with gentle rocking. The immune complexes were collected by the addition of 25 μL of protein A/G Magnetic Beads (Thermo Scientific), incubated for 1 h at RT and centrifuged. The precipitates were washed five times with 1x washing buffer (pH 7.4, 0.025 mol·L^−1^ Tris, 0.15 mol·L^−1^ NaCl, 0.001 mol·L^−1^ EDTA, 1% NP40, and 5% glycerin), and the immunoprecipitated complexes were suspended in loading buffer (pH 7.4, 0.025 mol·L^−1^ Tris, 0.15 mol·L^−1^ NaCl, 0.001 mol·L^−1^ EDTA, 1% NP40, and 50% glycerin) and subjected to SDS-PAGE and western blotting analyses using a Pinch1 or Smad2/3 antibody.

### Statistical analyses

The sample size for each experiment was determined based on our previous experience. Statistical analyses were performed using Prism GraphPad. Unpaired Student’s *t* test (two groups) and two-way ANOVA (multiple groups) followed by the Tukey–Kramer test were used for analysis. *P* < 0.05 was considered statistically significant.

## Supplementary information

Supplemental figure 1

Supplemental figure 2

## Data Availability

All data generated for this study are available from the corresponding authors upon reasonable request.
